# Development of a novel intervention using a person-based approach to support physical activity among families of children with cystic fibrosis in the UK

**DOI:** 10.1136/bmjopen-2024-093843

**Published:** 2025-10-23

**Authors:** Anastasiia G Kovalenko, Sarah Denford, Samantha van Beurden, Emma Cockcroft, Vicky Coxhead, Owen William Tomlinson, Emma Powell, Craig Anthony Williams

**Affiliations:** 1 University of Bristol Population Health Sciences, Bristol, UK; 2 Children’s Health and Exercise Research Centre, Department of Public Health and Sport Sciences, University of Exeter Faculty of Health and Life Sciences, Exeter, UK; 3 Health Protection Research Unit (HPRU) in Behavioural Science and Evaluation at the University of Bristol in collaboration with UK Health Security Agency, National Institute for Health and Care Research, London, UK; 4 Department of Health and Community Sciences, University of Exeter Medical School, Exeter, UK; 5 Unaffiliated Parent of Young Person with Cystic Fibrosis, UK, UK; 6 Academic Department of Respiratory Medicine, Royal Devon University Healthcare NHS Foundation Trust, Exeter, UK; 7 Department of Primary Initial Teacher Education, Faculty of Education, Birmingham Newman University, Birmingham, UK

**Keywords:** Cystic fibrosis, Behavior, Exercise, QUALITATIVE RESEARCH

## Abstract

**Abstract:**

**Objectives:**

Cystic fibrosis (CF) is an inherited condition, affecting approximately 150 000 people worldwide. Physical activity (PA) is an integral component in the management of CF. However, it is estimated that only a third of young people (with and without CF) achieve UK Chief Medical Officer guideline recommended levels of activity. The aim of this research was to use the person-based approach to develop an intervention supporting families with young people (aged 6–12 years) with CF to incorporate PA as a sustainable habit in their lives to increase the likelihood of sustained PA levels going into adolescence and adulthood.

**Design:**

Using the person-based approach, intervention content was created and iteratively adapted. This was initially guided by relevant literature; the guiding principles, logic model and preliminary content were developed via co-production with patient and public involvement (PPI) representatives (n=8) with lived experience of CF. The intervention was further refined/optimised using qualitative think-aloud and retrospective interviews, the results of the preliminary evaluation are reported. Think-aloud interviews were rapidly analysed using a table of changes analysis and used to inform adaptations to content. Retrospective interviews were analysed thematically.

**Setting:**

Community settings in the UK.

**Participants:**

Participants included six families with a child with CF aged between 6 years and 12 years old.

**Results:**

Intervention content consisted of nine sections and was delivered as a printable PDF file. Informed by the Capability, Opportunity, Motivation and Behaviour framework and self-determination theory, content focused on promotion of PA as a family activity that is fun, enjoyable, quick and achievable. It promoted ‘movement to make you feel good”’ and in short bursts of activity. Promotion of PA as medicine was avoided. The final intervention was considered to be engaging and acceptable.

**Conclusions:**

Qualitative methods and PPI facilitated the development of a family-focused intervention supporting the integration of PA into daily life. This was viewed as acceptable and engaging among families of people with CF. Future research now needs to explore the effectiveness of the intervention for increasing PA behaviour.

**STRENGTHS AND LIMITATIONS OF THIS STUDY**

- A key strength of this study is the use of a theory, evidence and person-based approach to co-produce an intervention to support physical activity among families of people with cystic fibrosis.
- Families with lived experience of cystic fibrosis were involved at all stages of the project.
- Due to challenges with recruitment, findings are based on a small number of interviews with families of people with cystic fibrosis.
- Some key voices may have been missing from the analysis (eg, those from minority ethnic communities).

## Introduction

 Cystic fibrosis (CF) is a genetically inherited condition, affecting approximately 11 000 people in the UK, and 150 000 worldwide.^1 2^ CF primarily affects the lungs and digestive system, causing thick mucus buildup, leading to infections and reduced lung function and eventual respiratory failure. With no cure, it is managed through nutritional, pharmacological, physiotherapy and exercise-based interventions.^3^

Exercise and physical activity (PA) (of which exercise is a structured subset) are recommended in CF management.^4^,^10^ PA (including exercise) has the potential to improve predictors of survival among people with CF (pwCF) (including aerobic capacity and lung function^11^,^14^), reduce symptoms of stress, depression and anxiety^15^ and increase quality of life.^12^ Guidance from the UK Chief Medical Officer states that healthy children and young people (5–18 years) should be moderately-to-vigorously active for at least 60 min per day, and guidance from the CF Trust^8^ stresses that this also applies to young pwCF. Despite the benefits, it is estimated that only a third of young people (with and without CF) achieve this recommendation.^16 17^

While attempts have been made to increase PA among pwCF,^18^ interventions have focused largely on the individual with CF, with little consideration given to the family and social context. Families can play an important role in supporting PA behaviour among children and adolescents;^10 19 20^ in particular, parents/carers can be strong role models for young pwCF,^21^,^23^ provide children with the skills they need to be active,^21^ provide tangible support,^24^ plan/structure activities, and support them to overcome barriers to activities.^21 23 24^ Moreover, parents/carers can provide opportunities, encouragement, and motivation for PA.^14 21 23^ Interventions with a high level of parental involvement are encouraged^25^ and are more likely to be effective for increasing PA behaviour than those targeting the young person alone.^2025^,^29^

However, research also indicates that families of pwCF often do not engage in PA themselves.^17 30^ In some cases, parents encourage their child to do PA as a treatment for their CF .^4 31^ This can result in the child feeling they have to be active; adding to the medicalisation of PA.^4^ This can reduce enjoyment and motivation for PA among young pwCF.^4^ To our knowledge, no interventions targeting young pwCF (under 12 years of age) alongside their families exist.

One promising approach for developing interventions is the ‘person-based approach’^32^—a systematic method of developing effective and engaging health behaviour change interventions. It uses indepth qualitative research, combined with relevant behavioural theory, to understand and accommodate the target population’s perspectives in the development of behaviour change interventions.^32^ The person-based approach is iterative, with continuous feedback used to adapt and refine the intervention, ensuring that the final intervention is engaging, acceptable and feasible for the target population.^32^

Therefore, the aim of this research was to use the person-based approach to develop and refine an intervention to promote PA among young pwCF (aged 6–12 years) and their families. In order to develop an age-appropriate intervention, we kept the age bracket as small as possible (6–12 years rather than 5–18 years). This allowed us to tailor content to the needs of the target population.

## Methods

**Figure 1:**
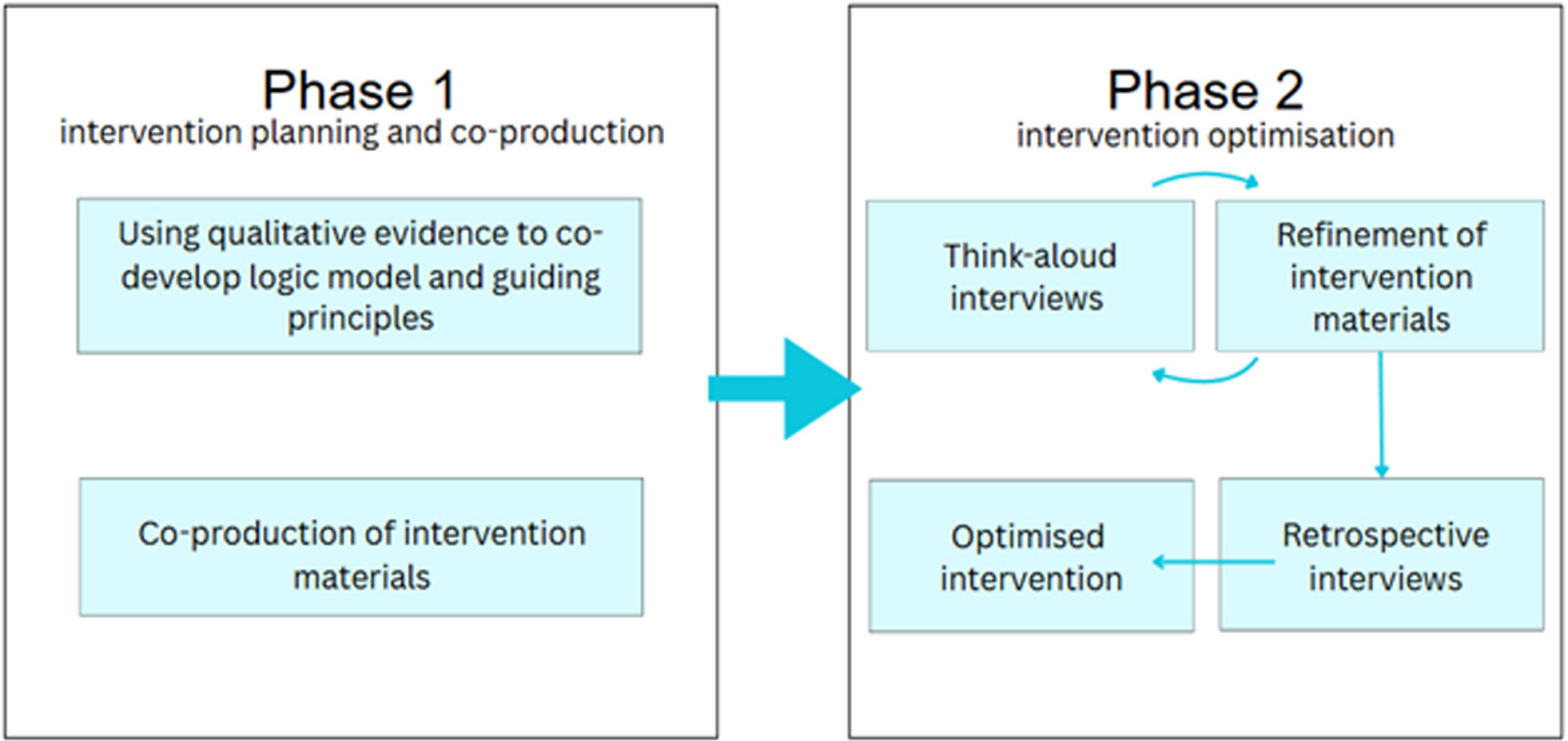
Study flowchart.

The intervention development comprised two phases (figure 1): (1) planning and (2) optimising of a PA intervention, as shown below:

1. Phase 1—intervention planning and co-production:
  - Patient and public involvement (PPI) and stakeholder engagement to inform the development of the guiding principles.
  - Developing a logic model and guiding principles.
  - Coproducing intervention materials.
2. Phase 2—intervention optimisation
  - Indepth think-aloud interviews.
  - Retrospective interviews.

### Patient and public involvement

A PPI group, recruited through social media and the researchers’ and collaborators’ existing contact database of people living with CF, was established at the beginning of this project. It consisted of eight families with young pwCF and met with the research team over six consecutive workshops. Attendance varied between five and eight PPI members at a time. Each session was prepared by the researcher (AK), with the focus of the meeting dependent on the stage of the project. The PPI group had two key roles: (1) to co-produce intervention content and (2) advise the study team on all aspects of the project. This included supporting the development of participant-facing materials and recruitment strategy, interpretation and dissemination of findings.

### Phase 1: intervention planning and co-production

#### Logic model

In line with the person-based approach,^32^ insight was gained from qualitative evidence,^33^ psychological and behaviour change theory and discussions with pwCF (PPI). Evidence from these sources was integrated to develop the logic model (a representation of how change in the intended outcome is to be achieved) and the guiding principles (the intervention features and design objectives addressing key issues crucial to engagement with the intervention for pwCF).

#### Guiding principles

Insights from the above were collated by the research team within a table of planning and used to develop the initial guiding principles.^32^ These identified and described context-specific characteristics are^4^ likely to influence engagement in PA, along with features for overcoming them. Guiding principles were developed by the research team, discussed with the PPI group and refined and finalised following feedback. The final set of guiding principles was consulted throughout intervention development to ensure it was underpinned by a clear and coherent focus.

#### Coproduction of intervention content/materials

Intervention content was coproduced through six workshops with the PPI group who were invited to contribute as equal partners within the research team. Initially, conversations were centred around an intervention planning table (online supplemental table S1), which outlined key barriers and facilitators, possible intervention features and associated behaviour change theory.^3234^,^36^

Barriers and facilitators to PA identified through previous literature^4 33^ were discussed and refined with the project team. The team then generated ideas for intervention content that could be useful for supporting engagement in PA among young pwCF and their families. Collaboratively, these ideas for content were prioritised and refined through discussion. Those considered unsafe, not feasible or problematic were removed. In line with the self-determination theory,^35^ the researchers were specifically concerned with identifying content to enhance autonomy, promote enjoyment, and promote movement within short windows of opportunity.

The research team mapped intervention content onto constructs from theoretical frameworks (eg, Capability, Opportunity, Motivation and Behaviour (COM-B) Model^34^) and specific behaviour change theory (eg, self-determination theory,^35^ social cognitive theory^37^). This process allowed us to identify any additional intervention techniques that may enhance the effectiveness.

### Phase 2: intervention optimisation

The aim of phase 2 activities was to use iterative qualitative research to ensure the intervention content and delivery was feasible, acceptable, engaging and persuasive,^38^ using think-aloud interviews and retrospective interviews.

#### Recruitment and ethics

Families were recruited through social media (Facebook, Twitter/X), promotion via the CF Trust and from our existing contact database of pwCF, who had previously consented to be contacted for research purposes. Project collaborators shared the recruitment poster (including a newsletter from a CF charity). Families were eligible to take part if they had at least one child aged 6–12 years who had a confirmed CF diagnosis and did not (self-report) meet guidelines of at least 60 min of moderate-to-vigorous PA per day and had access to a video-calling device. A £20 ‘thank-you’ payment was given to each family.

Once adaptations were made based on think-aloud interviews, a new set of families was recruited to use the intervention materials at home and take part in the retrospective interviews. No incentives were provided at this stage.

#### Data collection

The think-aloud interviews were done via Zoom at times that were mutually beneficial. The topic guide was codeveloped by the research team and the PPI group using a person-based approach and included three sections: (1) prethink-aloud; (2) think-aloud and researcher prompts and (3) post-think-aloud questions. The first section asked about types of PA young pwCF do and who they perform them with in everyday life, while the parents were asked about length of activity. The second section focused on prompting to talk about the intervention materials in real time. For example, ‘*What made you choose this activity? Can you tell me a bit more about why you think that?*’ The third section asked about the overall thoughts about the materials. For example, ‘*Which activities would you be willing to try? Why?*’ and ‘*Could you tell me about anything that would make it easier to try these?*’ The interviews were audio-recorded and transcribed verbatim. The interviewer (AGK) took field notes of any observed non-verbal cues and behaviours to clarify the context and note down reflections.

Retrospective interviews were then held with families following use of the refined intervention materials. The topic guide was developed in collaboration with the PPI group and aimed to explore participants’ thoughts and experiences of using the intervention in their day-to-day lives. Particular attention was paid to barriers to use, and changes in self-reported behavioural outcomes (eg, increased action planning). Sample questions: ‘*How easy or difficult was it to understand the instructions?*’ ‘*How did you find the process of planning the activities?*’ The interviews were conducted via Zoom, audio-recorded and transcribed verbatim.

The intended sample size was based on the concept of information power,^39^ which suggests the more informative the data set is, the smaller the sample required. Based on previous experience, we anticipated that about 15 interviews would be enough to provide rich data.

#### Procedures

##### Think-aloud interviews

All parents/carers received an information sheet outlining the study, and children were provided with an age-appropriate simplified version. After written consent and assent were received, participants took part in up to three think-aloud interviews during which they were encouraged to ‘think-aloud”’ while they were working their way through the intervention materials. Participants were instructed to open the file (a PDF document) on their device and share screen. They were asked to say out loud exactly what they were thinking while they worked through sections of the intervention. Each interview ended at the family’s request, which was approximately 55 min after beginning the interview.

##### Retrospective interviews

After written consent and assent were received, participating families were sent the refined intervention materials as a single PDF file by email and instructed to use it in their own time for 1 month. They were invited to take part in the interview 2–4 weeks following their month of using the intervention materials.

### Analysis

#### Think-aloud interviews

The transcripts were pragmatically coded for positive and negative comments about the intervention, tabulated in a table of changes and categorised following recommendations outlined by the person-based approach^32^ to facilitate prioritisation of changes to be made. Comments were categorised into: (a) important for behaviour change, (b) easy and uncontroversial, (c) frequently mentioned, (d) supported by experience (patients, carers or healthcare professionals), (e) contradicting experience or guiding principles, and finally (f) to be implemented.

#### Retrospective interviews

Interview transcripts were organised and analysed in NVivo V.12 software. Following the stages of reflexive thematic analysis,^40^ the researcher (AGK) read the transcripts and developed the preliminary codes. One interview was reviewed against the codebook by a second researcher (SD), followed by a discussion with the first researcher (AGK) and any discrepancies were resolved. The codebook was discussed with the wider research team (all co-authors). The researcher (AK) generated initial themes and checked against the dataset. Themes were then defined and named collaboratively with the research team (including PPI representatives) to sense-check and ensure that the findings were relevant.

## Results

### Phase 1: intervention planning and coproduction

#### PPI characteristics

Eight parents or guardians of children with CF aged 6–12 years old joined the PPI group. Parents took part in the workshops alone or in parent-child dyads.

#### Logic model

The logic model (online supplemental figure S1) addressed the three key needs outlined by the self-determination theory: autonomy, competence and relatedness and listed the intervention components targeting these needs and the short-term, medium-term and long-term outcomes of the planned intervention. The table of planning was incorporated into the guiding principles (table 1) and depicted key barriers and facilitators to engagement in PA among young pwCF and associated intervention features mapped to behaviour change theory.

**Table 1:** Summarised guiding principles

User context	Intervention design objectives	Key features/design
Due to complex and demanding medication regimes, young people with CF have limited time for PA. In this context of limited time” there is a lack of perceived importance of PA—with young people feeling the need to only partake in activities they enjoy. Younger children are less motivated by health benefits due to having fewer visible symptoms/seeing less immediate benefit of PA in terms of symptom reduction.	To support young people to engage in fun and enjoyable activities through offering users choice and autonomy regarding the content and activities they engage with to build intrinsic motivation.	Highlight benefits of physical activity for everyone. Building an enjoyable routine now to promote feeling better, autonomy, helping airway clearance, connection with the family, enhancing mood and general quality of life ahead. Avoid words such as ‘treatment’, ‘exercise’ or ‘physical activity’ that may have negative connotations. Prompt reflection on what the individual thinks is a valuable outcome (why would you want to be active?).
Engagement in PA can be scary and unpleasant due to breathlessness, coughing and sensations such as discomfort, muscle soreness, fatigue and joint pain. This can lead to doubts and concerns about the safety of engaging in activity.	To inform users that activities are safe for pwCF. To build confidence in engaging in PA and trying different activities and to encourage people to use strategies to prevent exacerbation of symptoms in an age-appropriate way. To support people, recognise the difference between sensations (such as breathlessness) that are normal and not normal during exercise.	Provide encouragement and reassurance for undertaking PA with CF provided in terms of CF-specific advice on consequences of activity, modelling examples of others with CF who have overcome barriers such as breathlessness and coughing during activity and links to peer support. Emphasise that users can reduce intensity and rest whenever necessary or avoid high intensity activity altogether. Provide information regarding what is ‘normal’ activity induced breathlessness and what is CF-related breathlessness (eg, it is normal to be out of breath after running—it is not normal to be out of breath sitting on the sofa, etc).
Levels of PA within the family are often low. Young people with CF feel singled out *when* they have to take part in activity and others do not.	To support engagement in enjoyable physical activities for all the family. To emphasise the benefits of PA in terms of physical and mental health for all.	Provide scientific information on the physical and mental health consequences of engaging in regular PA.

CF, cystic fibrosis; PA, physical activity.

#### Guiding principles

Intervention content had to focus on supporting families^24 33^ to build intrinsic motivation and identify and engage in activities they find fun and enjoyable. Content needed to highlight the immediate benefits of PA for people with and without chronic conditions, and avoid words such as ‘treatment,’ ‘exercise’ or ‘physical activity’ that may have negative connotations.^33^ Content needed to help users build confidence in PA and recognise the difference between sensations that are normal (eg, breathlessness) and not normal (eg, extreme breathlessness, pain) during PA. Table 1 provides a brief overview of guiding principles underpinning this research.

#### Intervention materials

The coproduced intervention was developed to address capability, opportunity and motivational barriers^34^ to PA as below and designed to be viewed online as a PDF. Sections could be printed out to act as physical reminders and prompts to action.

##### Capability

To address capability barriers, the section titled, ‘*Let’s find out why physical activity is important!*,’ provided information on positive health and emotional consequences of PA. It included a script of an animation of a human body with benefits of staying physically active. This was followed by videos and vignettes of relatable role models, created by the PPI group and other young pwCF (and their families). A videographer led the development of the films, conducted at locations familiar to the young person. During the films, young people discussed their motivations for taking part in PA, things they struggled with and recommendations for other pwCF. Conversation was interspersed with images of the young people taking part in PA they enjoy. This phase had additional support from the University of Exeter Engaged Researcher Award fund. The vignettes were coproduced with the PPI group and the research team. A list of frequently asked questions about symptoms, exacerbations and activity levels with answers was put together by the paediatrician on the research team (BE).

##### Motivation

Motivational barriers included content to encourage young people to identify enjoyable activities through games such as activity dice (player comes up with a list of activities and rolls the dice), challenge cards (player draws a card that challenges them to perform a fun activity) or the activity alphabet game (player comes up with an activity for each letter of the alphabet). These games were focused on encouraging creativity and autonomy and supporting young people to think beyond activities that they may have tried (and disliked) in the past. Content also included a table of ideas for fun activities to try out, as identified by the PPI group. Activities were grouped according to the likely physical effort levels (as agreed with the project team, who have extensive international expertise in paediatric exercise physiology) with an indication of time and resources needed.

##### Opportunity

To address opportunity barriers, content aimed to support incremental goal setting and progress tracking. The final section was entitled ‘*When your routine changes’*, consisting of a vignette and examples of activities appropriate for settings likely to disrupt routines, for example, holidays or inpatient admissions. This included encouragement to engage in short bursts of PA, a process frequently referred to as ‘exercise snacking^41^’, whereby people engage in several short bursts of PA spread throughout the day (rather than a single continuous session).

### Phase 2: intervention optimisation

#### Indepth qualitative think-aloud interviews

Three families with young pwCF took part in the interviews (ranging 57–86 min, mean duration was 73 min) between June and September 2021. Two families identified as white British and one Hispanic, all English-speaking. The age of children within each family was 5 years (about to turn 6 years), 9 years and 12 years. Participants described themselves as ‘not active’ (n=2) or ‘moderately active’ (n=1).

Overall impressions of the intervention were positive, with participants also identifying areas requiring additional work. The full table of changes highlighting positive and negative comments, features that required potential changes and decisions regarding changes is presented in online supplemental table S2.

##### Content

Changes relating to programme content were required to ensure age-appropriateness. For example, young people thought the language needed to be simpler, and role models needed to be of a similar age to the target audience. Goal setting sections needed to focus on setting family (rather than individual) goals. Additional clear and simple instructions with pictures were needed on how to perform activities. Families also thought that activities should be organised according to intensity levels, and intensity indicators should be explicitly stated. For example,

In terms of intensity and length… Is 10 minutes on the trampoline the same as running around for half an hour? I honestly do think videos are good. Even if the parents asking and then a doctor or a professional answering. (Mother of a 12-year old girl)

I was just going to say, some younger children, if you've got tubes and gastric buttons. They might not realise they can't do stuff. Maybe just say ‘please consult with your physician first, before engaging in contact sports’ or something just for everybody, not just kids with tubes. (Mother of a 12-year old girl)

**Figure 2:**
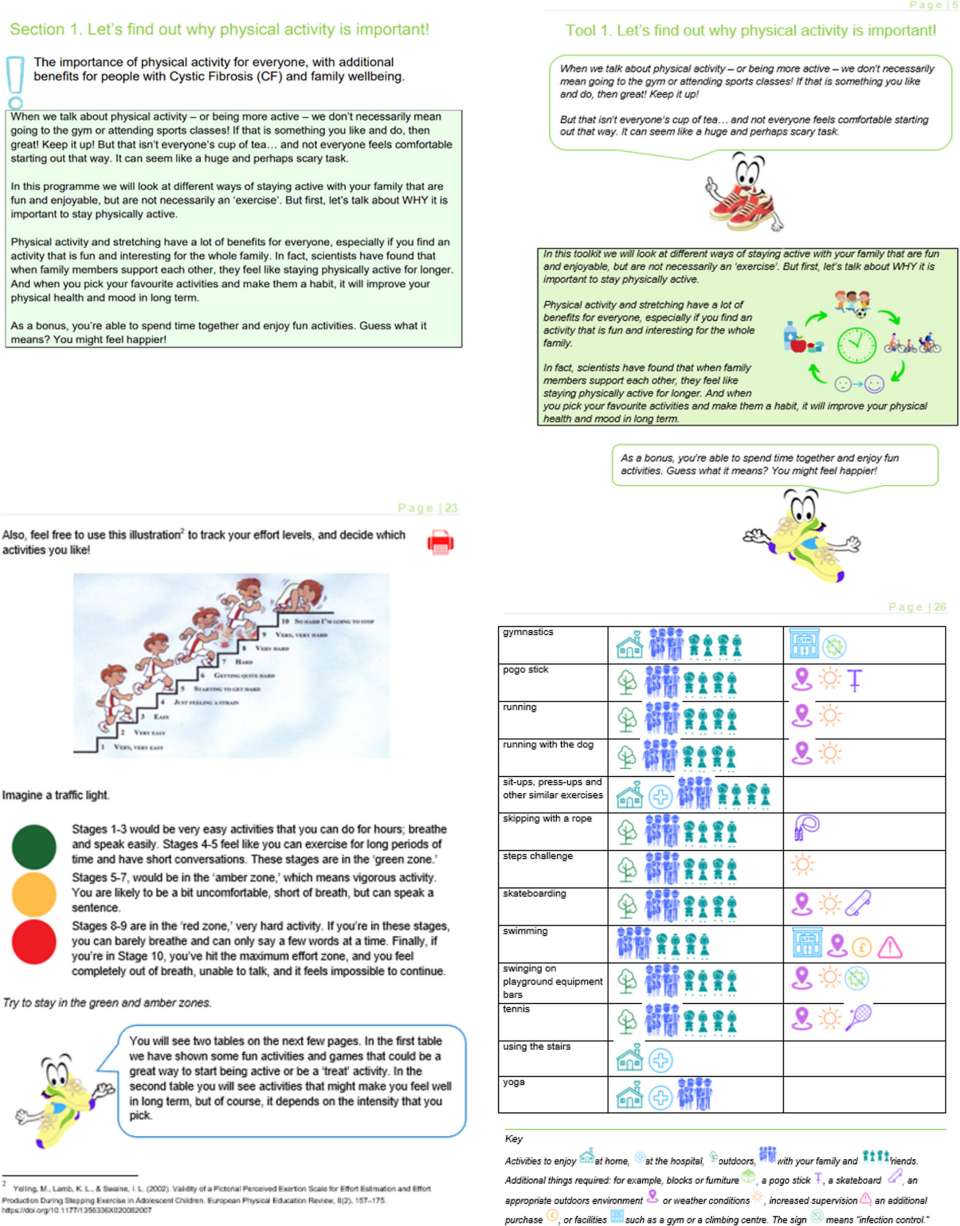
Intervention materials before think-aloud interviews (left) and after (right).

The Pictorial Children’s Effort Rating Table^42^ was used and the activities were reorganised by intensity levels. The framing of the materials was changed from ‘programme’ to ‘pick’n’mix toolkit’ to further promote ‘exercise snacking’ and facilitate the use of the materials (figure 2).

##### Design changes

Changes included the addition of age-appropriate and ethnically-diverse pictures throughout. For ease of navigation, quick response codes linking to videos were added. Printable sections were indicated (figure 2) to facilitate the use of track sheets and other materials which participants thought could be useful to have on hand (eg, table of activities).

### Retrospective interviews

The study did not recruit the anticipated number of participants (up to 15 interviews), in part due to the COVID-19 pandemic-related challenges, which were also the case in other studies at that time.^43^ The final decision was pragmatic, and recruitment ended when our recruitment window expired. The refined intervention materials were provided to four recruited families to use at home between June and October 2021. The ages of children were 6 years, 8 years, 10 years and 12 years (at the time of recruitment). One family did not take part in the subsequent interview due to CF-related health complications but was planning to use the materials at a later date. Three families took part in the interviews, 2–4 weeks following the month of using the intervention materials (September–December 2021). Two of these families used the intervention materials at home within the 1-month timeframe, and one family had started using the materials 1 week before the interview. The interviews ranged between 48 min and 49 min with a mean duration of 49 min.

#### Engagement with intervention materials

All participants used the intervention materials together: in parent-child dyads, as the whole family and in child-sibling dyads. In all families, the parent(s) read the document and/or skimmed through the pages first. Although none of the children read the intervention materials in full, they visited sections in the document (particularly section 2 *‘How many interesting activities can I think of?’* and section 4 *‘Ideas: here are some fun activities to try out’*).

#### Barriers to using the programme

Despite the aim to develop intervention content to support ‘exercise snacking’, a lack of time was still considered a barrier. Time was a particular issue during holidays, when families struggled to engage in any activities. For example, one family discussed how their activity schedule was disrupted during winter holidays.

You don’t keep sticking to [schedule]… I think we just got busy with Christmas. I think maybe now is a good time to re-pick it back up. (Mother of a 13-year-old girl).

Families mentioned that the absence of a hard copy of the materials provided by the research team could be restrictive and might have been a potential reason for limited engagement within the month of usage. Although two families were able to print the materials and try the activities.

And we’ve got [the activities] here… [lifts the laminated hard copy of the intervention materials] that went down well, didn’t it? You did it with your friend and your brother.’ (Mother of a 10 year old girl)

Families also noted that the amount of text may need to be reduced further or replaced with pictures/animations, to be more accessible to younger children:

Whether it needs to be broken down and maybe it’s 6–9 and 9–12 and a different kind of a pack for the two different age groups? (Mother of a 13-year-old girl)

#### Strengths of the programme

Families highlighted a number of strengths of the programme. In particular, games prompting players to create their own sets of activities and the generic list of activities with detailed instruction regarding resources and conditions required. For example,

It was good for motivating again and thinking about the stuff you can do inside in the winter; because you get into a habit of what you do in the summer outside, on the trampoline or whatever. You know you have to think again in the winter and you easily get bored so it’s good to get ideas to try to keep interested… (Mother of a 10-year-old girl)

[The intervention] has definitely brought our attention to trying to find more ways to get activities in. And it became a lot easier. So it’s definitely become more in our mind as to how to be more creative and how to engage back with activity stuff. (Mother of an 8-year-old boy)

Participants thought the content was clear and informed about ‘exercise snacking:”

Quite clear instructions on physical activity. And I like the fact that [the intervention] kept reminding you didn’t have to do all the exercise in one go. And I think we forget about that sometimes. (Mother of a 13-year-old girl)

All families expressed their willingness to continue using the materials, in particular the activities they perceived as ‘fun.’ These included games to think of appropriate activities, as well as the generic table listing the activities with instructions about the resources required.

In this game, I think they’re having the animal [parent shows the activity cube they made on a colour paper; the child laughs]. That made everybody laugh a lot, didn’t it? And it made it more silly and funny. I think that was the one you wanted to do the most at first. (Mother of a 10-year-old girl)

#### Potential impacts

Families thought taking part increased knowledge about impact and benefits of staying physically active, increased interest in trying out new activities and increased understanding of the importance of gradual exercise. There was also some evidence of autonomy, with participants actively selecting activities to try and engaging or planning to engage in selected activities with family and friends.

It’s easier now because she knows we’re not gonna make [her] do 50 burpees or anything like that. Sometimes if she’s tired then it’s kind of like, come on, let’s go for a walk or something… It’s more of a habit now I think. (Mother of a 13-year-old girl)

Obviously we'’ve increased activity since [recruitment]… [We’re] more aware of how important it is to try and find the right thing for him. (Mother of an 8-year-old boy)

## Discussion

This study described the process of the development of an intervention to promote PA among families of young pwCF and its preliminary evaluation. The Person-Based Approach^44^ was used to ensure that the intervention was both theoretically sound and engaging for the target population. Generally, the intervention content was considered acceptable, engaging and fun among families. However, there is a need to explore the acceptability and accessibility of the intervention with a larger, more diverse group of pwCF in the future.

The importance of behaviour change theory in intervention development is widely accepted.^45^ However, there is a lack of guidance regarding the translation of theory into intervention content. The person-based approach provides a systematic and transparent approach for coproducing content with target population. A systematic review of qualitative research^33^ provided insight into the barriers and facilitators to engagement in PA among young pwCF, and as a result, an intervention planning table enabled us to systematically list all barriers and facilitators, alongside possible intervention content and behaviour change theory. This ensured the intervention was grounded in theory and provided a foundation for the logic model underpinning the content.

In accordance with our, and others’ previous work,^33 46^ we used the COM-B framework to self-determination theory.^47^ In collaboration with PPI, the results of the review also informed the development of guiding principles, which describe contextual barriers to engagement in the intervention among young pwCF. This ensured the developed content was acceptable among the target audience.

A key barrier to engagement in PA among young pwCF is that there are few immediate perceived benefits.^33^ As a result, motivation to engage in the intervention content, and subsequently to engage in PA, was likely to be low. Intervention was therefore designed to be fun and enjoyable, encouraging people to identify activities they would enjoy and complete with the family. As it was important to avoid evoking negative connotations associated with PA—particularly as a treatment; instead, we focused on supporting ‘movement to make you feel good’. As young people often lack time in which to partake in activities,^48^ intervention content intended to support and promote PA in short bursts and in the home environment. Short bursts of activities to increase engagement in PA can be acceptable and accessible.^49^ The finding that children had picked activities they wanted to try, thought were fun and either engaged or were planning to engage in, shows that the intervention materials were used as intended.

Family engagement was also identified as being important, removing the focus on young pwCF and encouraging participation in PA as a normal part of maintaining a healthy active lifestyle.^27 50^ Despite evidence for the role of the family in supporting activity,^19 20^ interventions targeting PA among pwCF often overlook this aspect. The current intervention therefore focused on enhancing autonomy and pleasure in PA through suggestions for family-based activities and videos and stories that emphasised the importance of family support and provided positive role models for families. All young people used the intervention materials with their family, which also attests to the use of the materials as intended.

### Strengths and limitations

A key strength of this work is the critical involvement of theory, evidence and pwCF at all stages of the intervention development. This included regular consultations with families and young pwCF through PPI and qualitative research. This helped ensure that the perspectives of those who will be using the intervention were accommodated, an important factor if interventions are to be successful.^32^

The main limitation is the small number of participants recruited. This study was carried out during the COVID-19 pandemic and for pragmatic reasons (due to the increased burden on the NHS), it was decided not to proceed with recruitment via hospitals and GP surgeries. This resulted in limited options for recruitment and may have contributed to the small final sample. From previous experience, we anticipated conducting about 15 interviews but pragmatically had to stop when the recruitment window expired. Given the difficulty in recruiting, the sample may not have captured the views of less active pwCF, and participants may have been already quite motivated to engage in a PA intervention. Results of the study need to be interpreted in light of this. Conducting research that further refines the developed intervention through qualitative studies with a larger and more diverse sample of the target population would be beneficial. In addition, the study did not record participants’ medical status (eg, from databases or self-reported). It is also noteworthy that there was no objective assessment of PA. This data should be collected in further research to compare the differences in engagement with the intervention depending on their medical status and the existence of complications.

### Implications for future research

This research led to the development of an intervention to support PA among young pwCF and their families through promoting PA as fun, enjoyable and beneficial for all. One important direction is to assess the long-term effectiveness of the intervention in sustaining PA behaviours among families with pwCF. Measuring sustained behavioural change is essential to determine if the intervention can create lasting improvements in health outcomes, as the ultimate goal is to integrate PA as a habitual part of families’ lives. Additionally, it would be valuable to explore how specific intervention components—such as family-based activities, autonomy-enhancing strategies and fun-oriented games—impact the adherence to PA over time. Understanding which aspects are particularly motivating for different age groups or family structures could lead to more tailored and potentially efficacious interventions.

Future research with broader demographic representation would help validate the effectiveness across different socioeconomic, ethnic and geographic groups. Moreover, assessing the potential social benefits of the intervention, for example, how it influences family dynamics or promotes shared PA activities, could offer insights into its broader social implications. Finally, understanding barriers to sustained use, especially for families with lower initial motivation, will be crucial for refining the intervention and enhancing its reach.

Establishing the impact of PA interventions is a challenge, but essential if we are to provide an evidence base for the role of PA in the management of CF. While PA is known to be of importance to pwCF (as demonstrated by the James Lind Alliance priority setting partnership with pwCF), the greater focus on pharmacological interventions will likely make PA ‘second best’—particularly among those who are not currently active. However, in terms of clinical practice, the shift in emphasis from what was ‘activity restriction’ to now ‘activity promotion’ due to improved pharmacological interventions means more understanding is required as to how to support PA among pwCF and the role that clinical providers can play.

## Conclusion

This study involved the codevelopment of a theory, evidence and person-based intervention to promote PA with families of pwCF, where the intervention focused on promotion of PA for the whole family as fun, enjoyable and achievable. Intervention content was modified through engagement with PPI representatives, a series of think-aloud interviews and the final version was considered to largely be engaging and acceptable to those taking part. Some further recommendations were highlighted, such as adaptations to delivery format, age-appropriate text and more animations for younger children to enable equality in access. Future research needs to explore the effectiveness of this intervention in increasing PA behaviour.

## Supplementary material

10.1136/bmjopen-2024-093843 online supplemental file 1

## Data Availability

Data are available upon reasonable request.
