# Supplementary material for: Development of a novel intervention using a person-based approach to support physical activity among families of children with cystic fibrosis in the UK
Source: BMJ Open. 2025 Oct 23;15(10):e093843. doi: 10.1136/bmjopen-2024-093843 (PMC12557749; doi:10.1136/bmjopen-2024-093843)
Supplement: online supplemental file 1 [file bmjopen-15-10-s001.docx]

Supplementary material appendices.

Figure S1. Logic model of the intervention
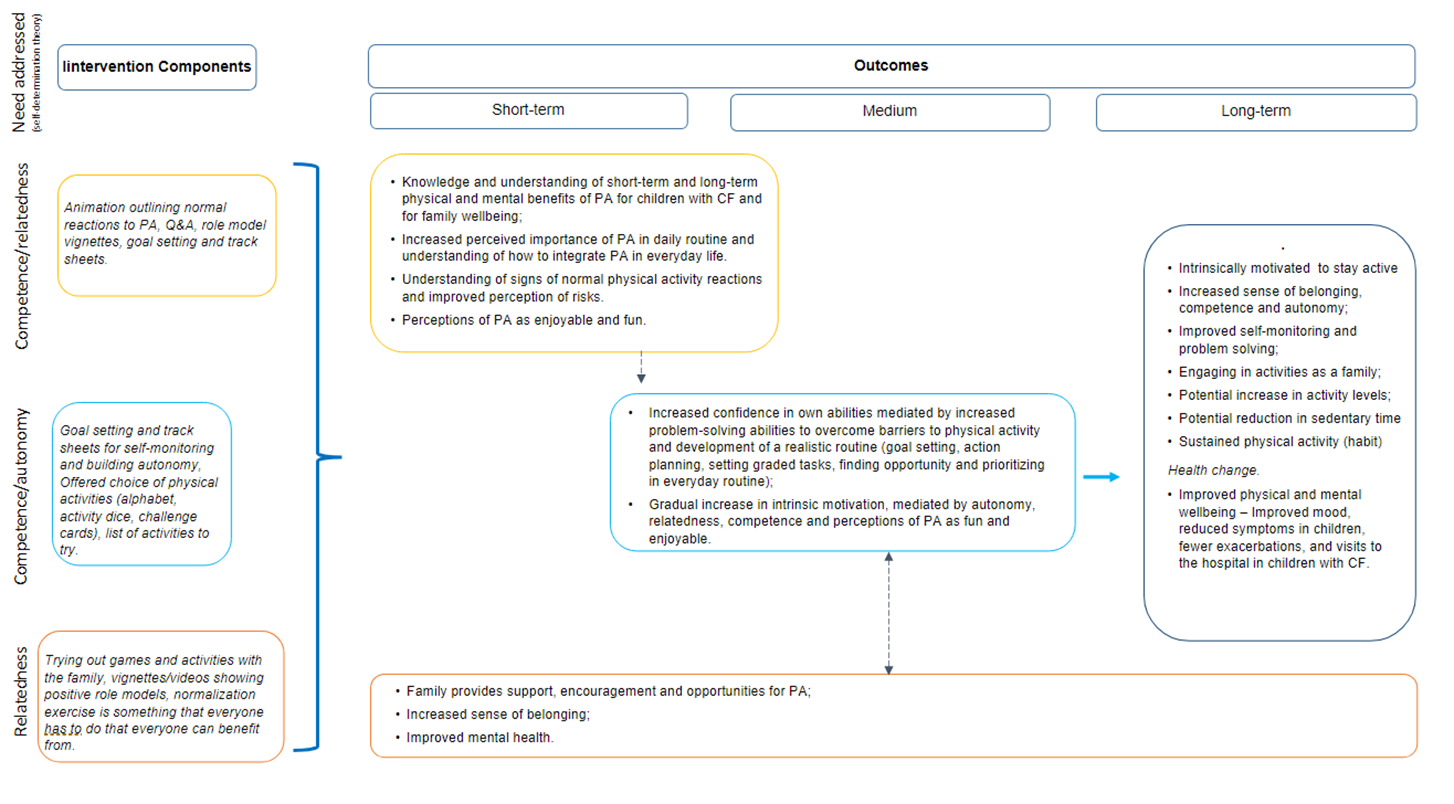


Table S1. Extract from Table of Planning.

| **Barriers** | **Potential facilitators (matched)** |
| --- | --- |
| Individual health conditions among parents may limit their participation in physical activity | Interactive approach  Educate about the benefits of physical activity, which might encourage to be physically active |
| Not encouraged by health benefits | Improve understanding of physical activity and be clear about why it is beneficial. |
| Limited time (also parents have to sacrifice their own time to ensure the child is physically active)  Reduced perception of importance of Physical Activity (PA) | Time-efficient activities  Potential incorporation with physio  Activities for the whole family. Family plays an important role in supporting children with Cystic Fibrosis (CF) |
| *Symptoms and Confidence*  Doubts and concerns of the parent about safety of their child during exercise | Individual approach: e.g. going for a walk, bicycle, dancing, boxing, trampoline  Age-appropriate activities |
| High levels of depression and anxiety among parents of children with CF  Isolation among parents | Connection with other families |
| Risk of cross-infection and being in isolation as a result | Activity should involve siblings or family members  Potentially have a competitive factor but not encourage rivalry. Competition as family units rather than individuals |
| Patients with CF can sometimes feel that they are different from their healthy peers | Reiterate normality: all children should be physically active |
| Patients with CF experience fluctuations in their health | Flexible activities and adaptable to change, especially in unexpected circumstances |
| *Environmental factors hinder participation*  Geographical boundaries might limit access to resources/activities. Seasonal changes impact the amount of physical activity. In the winter, a child is not as motivated to be active compared to in the warmer seasons. | - Tailored activities: urban vs countryside, home vs outdoors, summer vs winter. - Hygiene should be discussed. |

Table S2. Shortened Table of Changes

| ***Activity*** | ***Possible Change*** | ***Reason for change***  ***EAS (Easy and uncontroversial)***  ***NCON (does not contradict)***  ***NC (not changed)*** | ***Agreed change*** | ***MoScoW***  ***(Must do; Should do; Could do; Would Like to do)*** |
| --- | --- | --- | --- | --- |
| *Educational Animation* | More pictures throughout the intervention resources  Potentially cartoons/animation instead of text  Make it interactive  Address knowledge (what’s normal for this age and how it can be altered)  Give examples of some exercise in the animation.  Change what’s a “normal reaction during exercise” to “what happens in the organ (e.g. brain)” during exercise.  Information could be provided as audio and text.  Define any scientific terms used in clear language that a young child could understand.  Diverse characters throughout intervention | **EAS NCON**    **EAS, NCON**    **EAS, NCON**    **EAS, NCON**  **EAS, NCON**    **EAS, NCON**    **EAS, NCON**      **EAS, NCON** | **Add more pictures throughout the intervention**          **Paraphrase the text**        **Paraphrase in simple language**    **Provide diverse pictures** | **Should**    **Could**    **Must**    **Could**    **Must**  **Could**    **Must**      **Must** |
| *Frequently Asked Questions Q&A* | What is a normal reaction to physical activity in children with CF depending on *age & gender*  *Tips on hygiene, safe environment & cross-contamination*  Potentially provide information about heart rate zones  Add information about intensity and substitutions      FAQ potentially as an audio or picture, but text works fine as well    Rephrase questions to make them simpler:  What is considered optimal exercise levels? To How much, physical activity should my child do?  What’s a normal reaction?  How to deal with the symptoms of exacerbation?  Add question: How to track progress?    In answer to optimal exercise levels define moderate intensity physical activity and vigorous intensity physical activity in terms of heart rate. Make it clear that heart rate is only a rough estimation of intensity.  Make it clear that intensity of an activity will depend on the individual and that just being active is good. | **NCON**      **NC, NCON**    **NC**        **EAS NCON**      **EAS NCON**    **NC (people might not come back to the programme after following the link)**  **EAS, NCON**    **NC**    **NCON**      **NCON** | **Use the effort rating scale**      **Provide info on breathing etc. using children’s effort scale. Make it clear that PA should not be swapped for physio**    **Paraphrased the questions**            **Paraphrased answer.**      **Use the children’s effort rating scale instead**  **Add a disclaimer about talking to physio**      **Address in goal setting text and add a traffic light-style explanation on effort** | **Could**        **Could**      **Will not have, Could**    **Could**            **Could**      **Must**        **Should**      **Should** |
| *Role model videos* | Potentially subtitles to videos to facilitate understanding  Age of role models in the videos - 11-12  Role model videos and vignettes shouldn’t have any negative details that could frighten child | **Eas**  **Ncon**    **EAS NCON**  **EAS NCON** |  | **Should** |
| *Vignettes with case studies* | Potentially vignettes in a form of videos and cartoons  (although text content would indirectly develop other skills)  Get rid of any negative language that could scare children and stop them from exercising, throughout whole intervention (even mother of 10 year old didn’t think it was good for child to hear about too much gory details)  Add photos next to the vignettes.  Add a new story about goal setting and tracking from the parents’ perspective.    Change age of characters to 11/12 | **EAS NCON**    **EAS, NCON**      **EAS, NCON**    **NCON**    **EAS NCON** | **Toby’s story made into an animation**    **Paraphrased stories**      **Section added**        **Added photo next to Megans story**    **Added a goal setting vignette + Vicky agreed to do a video interview** | **Should**      **Must**    **Must**    **Should**    **Could**      **Should**    **Should** |
| *Challenge activity:*  *Alphabet* |  |  |  |  |
| *Challenge activity:*  *Activity dice* | The activity dice challenge can be altered (this is what the interviewee did) – cards instead of dice    Provide materials for challenge activities with intervention.  Say challenge activities could be done with other children | **EAS, NCON** |  | **Could** |
| *Challenge activity:*  Activity time |  |  |  |  |
| *Challenge activity:*  Activity bowl |  |  |  |  |
| *Challenge activity:*  Rolling the dice | Good to have a variety of activities.    Good to provide materials needed for activity so no preparation is required |  |  |  |
| *Goal setting activity* | Get rid of second goal.  Good to have a variety of goals as different people will be motivated by different things. | EAS NCON | **Edit text to remove all related to intensity. Instead use enjoyment and frequency**    **Edit text in all goal setting and tracking to address family activity rather than individual**    **traffic light-style explanation on symptoms during PA to describe intensity** | **Should**            **Must**              **Could** |
| *Tracking sheets*  *Comprehensive sheets* | Potentially change track sheets to family track sheets?  Potentially simplify track sheets to make them weekly or monthly  provide a variety of options.  Offer a variety of backgrounds, gender neutral    Check goal setting in young children literature.  Provide stickers to use on the track sheet.      Suggest that reflective section of track sheet could be done as a discussion.  Provide sheets alongside intervention | **NC, NCON**    **EAS**  **NCON**    **EAS NCON**    **EAS NCON**    **EAS NCON**    **EAS NCON**    **EAS NCON** | **Added in the goal setting vignette**  **Provide a track sheet that allows stickers or ticking boxes**    **Added discussion track sheets**    **Track sheets will come as an attachment** |  |
| *Tracking sheets*  Simpler version by week | Good to have a variety of activities. More comprehensive versions better for older children.  Create a simpler track-sheet for younger children.  Check goal setting in young children literature  Make it look more exciting (add colour) |  | **Provide a variety of track sheets (design and style-wise)**    **Change focus to family rather than individual in the track sheets** | **Should** |
| *Activity tracking*  Digital | Potentially encourage whole family to track their activity and make it a competition. |  |  |  |
| *Activity tracking*  A jar | Potentially suggest that different coloured items could be used to track different activities. |  |  |  |
| Activity: Walking stories |  |  |  |  |
| Activity:  Yoga | Add clear instructions on how to perform this exercise (from a specialist) |  |  |  |
| Activity:  Scavenger hunt | Provide paper scavenger hunt sheets with intervention materials.    Suggest that if they want to could split into teams to do it and win a reward. |  |  |  |
| Activity: Obstacle course |  |  |  |  |
| Instructions (important information before exercising) | Potentially instruction (water, sunscreen, medication etc.) in a form of pictures for younger children      Potentially create tables/charts with clear instructions by age (in terms of what’s normal, what’s recommended etc.) Maybe include in FAQ how much food water they should be taking in    Potentially modify language so that the children would not feel singled out | EAS NCON            **NC** | **Provide clear instruction with pictures**        **Instead, address this in goal setting explanation and effort rating scale** | **Must** |
| List of activities | Have a key for the icons rather than putting them in a paragraph to make it easier to follow.  Potentially split activities by intensity level.  Potentially encourage families to measure heart rate whilst trying the various activities so they have a rough estimate of the intensity of the activities.  Mention that not all nintendo switch games involve being physically active.      Add an infection control icon on anything involving balls  Make sure that the activities included in the table are equally appropriate for all genders | **EAS**  **NCON**      **NC** | **Put the key on each page with the table**          **Address in information, focus on effort rating scale and explain it is individual**      **Added infection control icon** |  |
| Staying active at the hospital | Potentially add information on hygiene and cleaning    Potentially add more questions and answers    Videos  Pictures |  |  |  |
| Other comments (general suggestions) | Ensure that language is easy to understand, and the instructions are clear    Potentially provide social support in a form of and online community of programme users.    Make it more geared towards the child by making the language more child friendly and encouraging the child to take ownership and make choices  Add more pictures throughout |  |  |  |
