# Supplementary material for: Development of a novel intervention using a person-based approach to support physical activity among families of children with cystic fibrosis in the UK
Source: BMJ Open. 2025 Oct 23;15(10):e093843. doi: 10.1136/bmjopen-2024-093843 (PMC12557749; doi:10.1136/bmjopen-2024-093843)
Supplement: Supplementary file 2 [file bmjopen-2024-093843.reviewer_comments.pdf]

PEER REVIEW HISTORY

BMJ Open publishes all reviews undertaken for accepted manuscripts. Reviewers are asked to complete a checklist review form and are provided with free text boxes to elaborate on their assessment. These free text comments are reproduced below.

ARTICLE DETAILS

Title (Provisional)

The development of a novel intervention using a person-based approach to support physical activity among families of children with cystic fibrosis in the UK

Authors

Kovalenko, Anastasiia G; Denford, Sarah; van Beurden, Samantha; Cockcroft, Emma; Coxhead, Vicky; Tomlinson, Owen William; Powell, Emma; Williams, Craig Anthony

---

VERSION 1 - REVIEW

**Reviewer**	**1**
**Name**	**Demeyer, Heleen**
**Affiliation**	**KU Leuven**
**Date**	**14-Feb-2025**
**COI**	**None**

I have read with great interest the paper "Supporting physical activity among families and children with cystic fibrosis: the development of a novel intervention using a person-based approach. The intervention development is very novel and in line with research and clinical needs in the present patient population. As indicated by the authors , the main limitation is the low number of families included in the study. I have some comments on the manuscript, mainly due to difficulties in interpreting the methods and results section.

1) Introduction:

- Even though the paper focuses on physical activity, the references used on the introduction are (also) relating to exercise capacity. The references supporting the statement “Physical activity (PA) is highly recommended in CF management [ref 4-8) are referring to the importance of exercise testing, not physical activity. I would recommend to focus more on paper showing the physical activity of patients with CF and avoid confusing with exercise capacity.

- In the description of the importance of family support, much evidence is based on qualitative data. I would recommend to also include Kinaupenne, J Cystic Fibros 2024,

adding insight into the importance of family support (or at least an active family member) based on objectively measured data.

- “In some cases, parents encourage their child to do PA as a treatment for their CF. This can result in the child feeling they have to be active; adding to the medicalisation of PA.” It not clear which evidence this statement is based on.

- In the introduction It is not clear why the authors focus on the age between 6-12 years. The age range is included in the methods section but not in the introduction.

2) Methods

- Which methodology did the authors use to develop the 2 phases described in the intervention development? Please add a reference.

- The authors use ‘co-production’ as well as ‘co-creation’. I would be consistent in the wording.

- “(2.2.2) Insights from the above were collated by the research team within a Table of planning and used to develop the initial Guiding principles. These identified and described context-specific characteristics likely to influence engagement in PA, along with features for overcoming them.” This is not clear to me. What do the authors mean with context-specific characteristics?

- “Barriers and facilitators to PA identified through previous literature were discussed and refined with the project team”. Which previous literature was used? Please refer.

- The authors excluded families with a child that would meet the PA guidelines (based on self-report). Couldn’t it have been interesting to include the entire spectrum of PA to discuss barriers and enablers, even though they would meet the guidelines? Self-reported PA has the risk to be an overestimation so you might have lost some inactive children.

- The methods section is not very easy to follow. Would it be possible to present the flow graphically?

Results

- The results section misses characteristics of the PPI (phase 1)? How many people were included? Are these all patients? This is not very clear to me.

- The results section misses characteristics of patients and caregivers (phase 2) included. Also a flowchart to show how many families were invited and included. In this especially the medication of CF patients (ITE?) is important.

- In the description of the guiding principles it is not clear to me how the ‘user context’ was decided. Also the intervention design objectives and key features are not very clear to me. Are these based on quotes of participants?

- The results section (3.1.2) includes references to previous literature. It is not clear why these are included. What information is based on new results collected? To me it seems more logic that knowledge from previous research are included in the methods section.

- 3.1.3.1: ‘a script of an animation of a human body with benefits of exercise’ . Do the authors refer to exercise of broader physical activity?

- P10 line 243: I don’t think it is needed to specify the funding in the paper in the results section.

Discussion

- The main limitation of the study is the small number of included families. In addition, one family only started the intervention one week before the interview. I would also add this as a limitation. To me it is not clear how many families were planned to be included.

- I think it is a limitation that the PA level of the included children is not known. The authors expect these might be representative and already motivated but this is difficult to interpret lacking data on PA.

**Reviewer**	**2**
**Name**	**Wojsyk-Banaszak, Irena**
**Affiliation**	**Poznan University of Medical Sciences**
**Date**	**10-Apr-2025**
**COI**	**None**

It is with great interest that I read the article: “Supporting physical activity among families of children with cystic fibrosis: The development of a novel intervention using a Person-Based approach” by Kovalenko and colleagues. Physical activity is an important part of everyday life and all children and adolescents should be encouraged to spend their time actively. It is even more important in children with chronic conditions including cystic fibrosis.

The paper is generally well-written and concerns an important topic. It has an additional practical value in describing an intervention that may encourage whole families to a more active lifestyle. There are some concerns however that need addressing before the paper can be recommended for publication:

1. The number of participating families (3 in total) is low. Cystic fibrosis is a rare disease, but even so, with such low numbers, there is a risk of bias towards families who are already into healthy lifestyle and who are interested in being more active (as the Authors state themselves). The Authors acknowledge that no voices from minority communities were included, but my worry would also be the generally low numbers of all voices. Taking this

into account I would suggest treat the paper as a preliminary report and validate the results on a larger group of pwCF in the future.

2. The clinical characteristics of participating children should be given. What were their lung function and general status, e.g. FEV1, BMI? Did they receive modulators, the drugs that are known to be changing the life of pwCF tremendously? It would also be interesting to have a clinical characteristic and age of the PPI

3. In the “Intervention planning and co-production” section (page 5), more details on “key barriers and facilitators, possible intervention features, and associated behavior change theory” would enhance the paper’s value

4. Recruitment part: How many families were actually contacted and declined? This should be mentioned for both phases of the experiment

---

VERSION 1 - AUTHOR RESPONSE

Reviewer: 1
Dr. Heleen Demeyer, KU Leuven
Comments to the Author:
I have read with great interest the paper "Supporting physical activity among families and children with cystic fibrosis: the development of a novel intervention using a person-based approach. The intervention development is very novel and in line with research and clinical needs in the present patient population. As indicated by the authors , the main limitation is the low number of families included in the study. I have some comments on the manuscript, mainly due to difficulties in interpreting the methods and results section.

Author reply:
We thank Reviewer 1 for the thorough review and insightful comments. We believe that addressing your questions and concerns will help us strengthen the manuscript.

1) Introduction:
- Even though the paper focuses on physical activity, the references used on the introduction are (also) relating to exercise capacity. The references supporting the statement “Physical activity (PA) is highly recommended in CF management [ref 4-8) are referring to the importance of exercise testing, not physical activity. I would recommend to focus more on paper showing the physical activity of patients with CF and avoid confusing with exercise capacity.

Author reply:

Thank you for this comment. It is indeed important to make sure that the wording we use in the paper is consistent
Physical Activity is the umbrella term for any bodily movement and thus will encompass sport, recreation, exercise albeit with slightly different aims to this movement [see Caspersen, C. J., Powell, K. E., & Christenson, G. M. (1985)]. We have now clarified this throughout the text, using “physical activity” throughout. This is except the term “exercise snacking” [37] as this is the established concept we referred to.

- In the description of the importance of family support, much evidence is based on qualitative data. I would recommend to also include Kinaupenne, J Cystic Fibros 2024, adding insight into the importance of family support (or at least an active family member) based on objectively measured data.

Author reply:
Thank you for this. The paper was published after we submitted for review, but we believe that our manuscript would benefit from citing this research. We therefore cite it on page 3

- “In some cases, parents encourage their child to do PA as a treatment for their CF. This can result in the child feeling they have to be active; adding to the medicalisation of PA.” It not clear which evidence this statement is based on.

Author reply:
Thank you. We have now clarified this by adding the citations on page 3

- In the introduction It is not clear why the authors focus on the age between 6-12 years. The age range is included in the methods section but not in the introduction.

Author reply:
We now clarify in the introduction that the focus of the project is on those between the ages of 6 and 12. Guidelines have been developed for children between the ages of 5 and 18 years. In order to develop an age appropriate intervention, we kept the age bracket as small as possible. This allowed us to tailored content to the needs of the target population. Our team have also developed content for older children (over the age of 12).

2) Methods
- Which methodology did the authors use to develop the 2 phases described in the intervention development? Please add a reference.

Author reply:
Thank you . We have now added the citations on pages 4 and 5.

- The authors use ‘co-production’ as well as ‘co-creation’. I would be consistent in the wording.

Author reply:
Thank you for this comment. Indeed, it is essential to use one term and be consistent across the manuscript. We have chosen to use “co-production” as this term reflects the work that was done, and have updated on page 5.
https://www.phrp.com.au/issues/june-2022-volume-32-issue-2/co-creation-co-design-co-production-for-public-health-a-perspective-on-definition-and-distinctions/

- “(2.2.2) Insights from the above were collated by the research team within a Table of planning and used to develop the initial Guiding principles. These identified and described context-specific characteristics likely to influence engagement in PA, along with features for overcoming them.” This is not clear to me. What do the authors mean with context-specific characteristics?

Author reply:
Thank you. In this case we mean contextual factors. We have now added a citation which we also utilised during the development of the guiding principles (Denford et al, 2020), page 5 lines 120-121

- “Barriers and facilitators to PA identified through previous literature were discussed and refined with the project team”. Which previous literature was used? Please refer.

Author reply:
We have now added the reference on page 5 lines 120-121.

- The authors excluded families with a child that would meet the PA guidelines (based on self-report). Couldn’t it have been interesting to include the entire spectrum of PA to discuss barriers and enablers, even though they would meet the guidelines? Self-reported PA has the risk to be an overestimation so you might have lost some inactive children.

Author reply:
Thank you. We acknowledge that it would have been interesting to include those who are active and meet the PA guidelines, but we were actively searching for the harder to reach groups – those who generally do not take part. The issue with recruiting in studies like this is that it’s always those who are already active who are interested in taking part.

- The methods section is not very easy to follow. Would it be possible to present the flow graphically?

Author reply:

Yes, thank you for this comment, we agree that it would clarify the method. We have now added it on page 4.

Results

- The results section misses characteristics of the PPI (phase 1)? How many people were included? Are these all patients? This is not very clear to me.

Author reply:

We have now added the characteristics on page 8

- The results section misses characteristics of patients and caregivers (phase 2) included. Also a flowchart to show how many families were invited and included. In this especially the medication of CF patients (ITE?) is important.

Author reply:

Thank you. Characteristics of patients and caregivers are provided in page 10, lines 277-281. These were the only characteristics recorded.

Unfortunately it is difficult to know how many were invited as all those who were interested in taking part contacted us after having seen the posts/targeted ads on social media. Overall, we received 3 expressions of interest to take part in the think-aloud interviews after posting on Twitter, Facebook, and in CF Foundation groups.

- In the description of the guiding principles it is not clear to me how the 'user context' was decided. Also the intervention design objectives and key features are not very clear to me. Are these based on quotes of participants?

Author reply:

Guiding principles specify specific user characteristics likely to affect engagement, such as their views, needs and capabilities, key intervention design objectives to improve engagement, and key features of the intervention that will achieve those objectives. They are developed based on existing qualitative research (e.g., Denford et al., 2020), consultation with stakeholders (including the project team, clinical collaborators, behaviour change experts, CF physiologists, physiotherapists, clinical psychologists and nurses) and co-produced with the PPIE group (see page 5 lines 119-143).

- The results section (3.1.2) includes references to previous literature. It is not clear why these are included. What information is based on new results collected? To me it seems more logic that knowledge from previous research are included in the methods section

Author reply:
Thank you for your comment. We decided that Phase 1 (co-production) fits into results, where provide in-depth information on the process and results. Therefore the references (which are similar to the ones used in the methodology) are cited to clarify which literature this was drawn on.

- 3.1.3.1: 'a script of an animation of a human body with benefits of exercise'. Do the authors refer to exercise of broader physical activity?

Author reply:
This means broader physical activity. We have now paraphrased this on page 10.

- P10 line 243: I don't think it is needed to specify the funding in the paper in the results section.

Discussion
- The main limitation of the study is the small number of included families. In addition, one family only started the intervention one week before the interview. I would also add this as a limitation. To me it is not clear how many families were planned to be included.

Author reply:
Thank you for the comment. The intended sample would have been informed by the concept of information power - but we had to be pragmatic and stop recruitment when we ran out of time. We have now added a statement in the limitations.

- I think it is a limitation that the PA level of the included children is not known. The authors expect these might be representative and already motivated but this is difficult to interpret lacking data on PA.

Author reply:
Thank you for the comment. It is known (the families self-reported being not active/not meeting the guidelines or being moderately active (see page 10).

Formatted: Font: (Default) Aptos, Complex Script Font: Arial, (Asian) Japanese, Ligatures: None

Formatted: Font: (Default) Aptos, Complex Script Font: Arial, (Asian) Japanese, Ligatures: None

Reviewer: 2
Dr. Irena Wojsyk-Banaszak, Poznan University of Medical Sciences

Comments to the Author:
It is with great interest that I read the article: "Supporting physical activity among families of children with cystic fibrosis: The development of a novel intervention using a Person-Based approach" by Kovalenko and colleagues. Physical activity is an important part of everyday life and all children and adolescents should be encouraged to spend their time actively. It is even more important in children with chronic conditions including cystic fibrosis.

The paper is generally well-written and concerns an important topic. It has an additional practical value in describing an intervention that may encourage whole families to a more active lifestyle. There are some concerns however that need addressing before the paper can be recommended for publication:

Author reply:
Thank you for the insightful comments. We believe that addressing your questions and concerns will help us strengthen the manuscript.

1. The number of participating families (3 in total) is low. Cystic fibrosis is a rare disease, but even so, with such low numbers, there is a risk of bias towards families who are already into healthy lifestyle and who are interested in being more active (as the Authors state themselves). The Authors acknowledge that no voices from minority communities were included, but my worry would also be the generally low numbers of all voices. Taking this into account I would suggest treat the paper as a preliminary report and validate the results on a larger group of pwCF in the future.

Author reply:
Thank you. We agree and think it may strengthen a follow-on funding application for further qualitative work. We have now paraphrased page 14 in the discussion.
In terms of diversity, we did have voices from minority communities (out of 6 families 1 was of Hispanic descent), but we do acknowledge that our sample might not have been representative.

2. The clinical characteristics of participating children should be given. What were their lung function and general status, e.g. FEV1, BMI? Did they receive modulators, the drugs that are known to be changing the life of pwCF tremendously? It would also be interesting to have a clinical characteristic and age of the PPI

Author reply:
We did not collect clinical data in this study and agree that it would be useful to examine these characteristics, in the next evaluation.

3. In the “Intervention planning and co-production” section (page 5), more details on “key barriers and facilitators, possible intervention features, and associated behavior change theory” would enhance the paper’s value

Author reply:
Thank you. We have now added a summary from the intervention planning table in Table S3 (supplementary).

4. Recruitment part: How many families were actually contacted and declined? This should be mentioned for both phases of the experiment

Author reply:
Thank you. Unfortunately this is difficult to know as all those who were interested in taking part contacted us after having seen the posts/targeted ads on social media. Overall, we received 3 expressions of interest to take part in the think-aloud interviews after posting on Twitter, Facebook, and in CF Foundation groups.

VERSION 2 - REVIEW

**Reviewer**	**1**
**Name**	**Demeyer, Heleen**
**Affiliation**	**KU Leuven**
**Date**	**30-Jun-2025**
**COI**	

I would like to thank the authors for providing answers to my review comments. I do have some remaining comments based on their rebuttal.

- I do agree with the authors that physical activity is an umbrella term, also including exercise. However, as the evidence is mainly based on exercise, this needs to be more clearly explained in the introduction.

- In their answer to my comment, the authors describe the rationale to focus on the age 6-12year. I would recommend to add this information in the manuscript.

- In their answer, the authors indicated that the sample size was planned to be based on information power. I would include this in the discussion section where the sample size is described.

- The authors answered that the PA level of the children is known. I would still include the lack of an objective assessment of PA as a limitation of the study.

**Reviewer**	**2**
**Name**	**Wojsyk-Banaszak, Irena**
**Affiliation**	**Poznan University of Medical Sciences**
**Date**	**17-Jun-2025**
**COI**	

The Authors have considerably improved their work. Unfortunately, there remain some issues that need addressing.

Regretfully, only a few of my concerns regarding this article, included in the first review, were properly addressed.

I asked how many patients were contacted and consented/declined. In the paper, the Authors state they used "social media and the researchers' existing database of people living with CF". Yet in their response to the Review, the Authors state that the Families contacted them on social media, so they cannot provide these data. If the Authors used the database, how many CF individuals in the interesting age range did it include? If the patients indeed responded to the social media ad, it would bias the participants towards those actively seeking more physical exercise and would, at least partly, explain the low participation rate.

I would still encourage the Authors to use "preliminary report" in the title of a paper that is based on results acquired from only 3 subjects.

It would be interesting to learn about the patients' general condition. These data should be possible to obtain from medical records (especially when the Authors have a database). I would imagine that it is much easier to engage in physical exercise for someone with good lung function and no complications as opposed to someone with chronic respiratory insufficiency.

All the abbreviations in the Document (including tables in the Supplementary files) should be clearly explained.

VERSION 2 - AUTHOR RESPONSE

Reviewer: 2

Dr. Irena Wojsyk-Banaszak, Poznan University of Medical Sciences

Comments to the Author:

The Authors have considerably improved their work. Unfortunately, there remain some issues that need addressing.

Regretfully, only a few of my concerns regarding this article, included in the first review, were properly addressed.

I asked how many patients were contacted and consented/declined. In the paper, the Authors state they used "social media and the researchers' existing database of people living with CF". Yet in their response to the Review, the Authors state that the Families contacted them on social media, so they cannot provide these data. If the Authors used the database, how many CF individuals in the interesting age range did it include? If the patients indeed responded to the social media ad, it would bias the participants towards those actively seeking more physical exercise and would, at least partly, explain the low participation rate.

Author Reply:

Thank you for your comment. The collaborators shared the ad with their contact database and newsletter subscribers – unfortunately we did not record the number of subscribers (clarified in page 6, lines 152-154).
Yes, the people with CF responded to the social media ad and we discuss the bias in page 16, line 439-447.

I would still encourage the Authors to use "preliminary report" in the title of a paper that is based on results acquired from only 3 subjects.

Author Reply:

Thank you for the comment. Development was based on considerable PPIE work and literature and is not a preliminary work. However, the evaluation can be called preliminary. Therefore we have now written "preliminary evaluation" in the abstract, p.1 line 18-19; and page 14 line 384 - discussion.

It would be interesting to learn about the patients' general condition. These data should be possible to obtain from medical records (especially when the Authors have a database). I would imagine that it is much easier to engage in physical exercise for someone with good lung function and no complications as opposed to someone with chronic respiratory insufficiency.

Author Reply:

There might have been confusion in terms the term "database", therefore we have changed the text throughout the manuscript to clarify that this was a database of existing contacts interested in taking part in further research (page 6 line 156) - and we asked our collaborators to share the ad. We did not collect the medical record data (e.g., from the GP or hospital), as we were not ethically permitted – and therefore acknowledge this as another limitation which we have now added on p.16 line 452-456:

In addition, the study did not record participants' medical status (e.g., from databases or self-reported). This data should be collected in further research to compare the differences in engagement with the intervention depending on their medical status and the existence of complications.

All the abbreviations in the Document (including tables in the Supplementary files) should be clearly explained.

Author Reply:

We have now updated the Supplementary document by putting:

National Institute for Health and Care Research (NIHR) on page 2; Physical Activity (PA) on page 4; Cystic Fibrosis (CF);

And in the table on page 4:

EAS (Easy and uncontroversial)
NCON (does not contradict)
NC (not changed)
MoScoW (Must do; Should do; Could do; Would Like to do)

Reviewer: 1
Dr. Heleen Demeyer, KU Leuven
Comments to the Author:
I would like to thank the authors for providing answers to my review comments. I do have some remaining comments based on their rebuttal.

- I do agree with the authors that physical activity is an umbrella term, also including exercise. However, as the evidence is mainly based on exercise, this needs to be more clearly explained in the introduction.

Author reply:

Thank you for the comment. We have now addressed this on page 3, line 53-54:

"Exercise and physical activity (of which exercise is a structured subset) are…"

And added citations 8-10 (line 55); and 14 (line 57)

Line 60 (removed "associated with PA").

- In their answer to my comment, the authors describe the rationale to focus on the age 6-12year. I would recommend to add this information in the manuscript.

Author reply:

Thank you. We have now added this on page 4, lines 89-90.

- In their answer, the authors indicated that the sample size was planned to be based on information power. I would include this in the discussion section where the sample size is described.

Author reply:

Thank you, this has now been added on page 7, lines 189-193; and in discussion page 15, lines 452-456:
"From previous experience we anticipated to conduct about 15 interviews, but pragmatically had to stop when the recruitment window expired."

- The authors answered that the PA level of the children is known. I would still include the lack of an objective assessment of PA as a limitation of the study.

Author reply:

Thank you, this has now been added on page 15, line 452-453, along with the reply to Reviewer 2's comment:

In addition, the study did not record participants' medical status (e.g., from databases or self-reported). It is also noteworthy that there was no objective assessment of PA. This data should be collected in further research to compare the differences in engagement with the intervention depending on their medical status and the existence of complications.

VERSION 3 - REVIEW

**Reviewer**	**1**
**Name**	**Demeyer, Heleen**
**Affiliation**	**KU Leuven**
**Date**	**26-Aug-2025**
**COI**	

I would like to thank the authors for providing an answer to my questions and changing the manuscript accordingly. I don't have any further comments.

**Reviewer**	**2**
**Name**	**Wojsyk-Banaszak, Irena**
**Affiliation**	**Poznan University of Medical Sciences**
**Date**	**08-Sep-2025**
**COI**	

The Authors have improved their work, which is now adequate for publication.
